# The mitotic spindle-related seven-gene predicts the prognosis and immune microenvironment of lung adenocarcinoma

**DOI:** 10.1007/s00432-023-04906-9

**Published:** 2023-06-02

**Authors:** Ruxin Shen, Zhaoshui Li, Xiaoting Wu

**Affiliations:** 1 Department of Thoracic Surgery, Affiliated Nantong Hospital of Shanghai University, Nantong, 226000 Jiangsu China; 2 Qingdao Medical College, Qingdao University, Qingdao, 266023 China; 3 Department of Radiation Oncology, Shanghai Pulmonary Hospital, Tongji University School of Medicine, Shanghai, 200433 China

**Keywords:** Lung adenocarcinoma, Mitotic spindle, Nomogram, Prognosis signature, Immune microenvironment

## Abstract

**Purpose:**

Abnormalities in the mitotic spindle have been linked to a variety of cancers. Data on their role in the onset, progression, and treatment of lung adenocarcinoma (LUAD) need to be explored.

**Methods:**

The data were retrieved from The Cancer Genome Atlas (TCGA), Gene Expression Omnibus (GEO), and Molecular Signatures Database (MSigDB), for the training cohort, external validation cohort, and the hallmark mitotic spindle gene set, respectively. Mitotic spindle genes linked to LUAD prognosis were identified and intersected with differentially expressed up-regulated genes in the training cohort. Nomogram prediction models were built based on least absolute shrinkage and selection operator (LASSO) regression, univariate cox, and multivariate cox analyses. The seven-gene immunological score was examined, as well as the correlation of immune checkpoints. The DLGAP5 and KIF15 expression in BEAS-2B, A549, H1299, H1975, and PC-9 cell lines was validated with western blot (WB).

**Results:**

A total of 965 differentially expressed up-regulated genes in the training cohort intersected with 51 mitotic spindle genes associated with LUAD prognosis. Finally, the seven-gene risk score was determined and integrated with clinical characteristics to construct the nomogram model. Immune cell correlation analysis revealed a negative correlation between seven-gene expression with B cell, endothelial cell (excluding LMNB1), and T cell CD8 + (*p < *0.05). However, the seven-gene expression was positively correlated with multiple immune checkpoints (*p < *0.05). The expression of DLGAP5 and KIF15 were significantly higher in A549, H1299, H1975, and PC-9 cell lines than that in BEAS-2B cell line.

**Conclusion:**

High expression of the seven genes is positively correlated with poor prognosis of LUAD, and these genes are promising as prospective immunotherapy targets.

## Introduction

The diagnosis and treatment of lung cancer (LC) have received a considerable deal of attention due to its high morbidity and mortality. Non-small cell lung cancer (NSCLC) accounts for more than 80% of lung cancers. Lung adenocarcinoma (LUAD) is the most common subtype of NSCLC (Bray et al. 2018).

The overall survival (OS) of LUAD patients has increased because of the use of targeted therapy and immunotherapy. Immune checkpoint inhibitors (ICIs) are extremely beneficial in patients with a negative driver mutation. However, drug resistance to ICIs is a serious problem that must not be overlooked. Current predictive indicators of responsiveness to checkpoint inhibitors include programmed cell death 1 (PD-1) and programmed cell death-Ligand 1 (PD-L1). More and more prospective or prognostic relevant target genes have been discovered through data mining. As such, one of the hotspots of current research is to develop a prediction model using bioinformatics for the individualized treatment of LUAD patients.

A spindle is a cellular structure that forms during mitosis or meiosis in eukaryotic cells and is mainly composed of microtubules, molecular motors, and other proteins (Pavin and Tolic 2016). The mitotic spindle regulates the correct chromosome segregation and any faults can cause aneuploidy and disease (Webster and Schuh 2017; Santaguida and Amon 2015; Potapova and Gorbsky 2017). These events may be linked to tumorigenesis (Levine and Holland 2018). Li and colleagues discovered that genes linked to chromosomal segregation predicted outcomes in LUAD patients (Li et al. 2022b). Thaiparambil et al. discovered an overlap in the expression of several mitotic spindle abnormalities and chromosomal mis-segregation-associated genes (such as AURKA, AURKB, and MAD212) in chronic obstructive pulmonary disease (COPD) and LC (Thaiparambil et al. 2020). Furthermore, studies on the use of mitotic spindle apparatus antibody (MSA) in small cell lung cancer (SCLC) diagnosis have also been published (Wu et al. 2018; Liu et al. 2022b).

There have been few investigations on prognostic models and mitotic spindle biomarkers, and the mechanism of LUAD by which the mitotic spindle influences chromosomal segregation remains unclear. In this view, we retrieved data on mitotic spindle-related gene sets from the Molecular Signatures Database (MSigDB) (Mootha et al. 2003; Subramanian et al. 2005) and performed correlation analysis with the prognosis of LUAD at different pathological stages, and constructed a prognostic model. The hub genes screened were evaluated for OS, immune microenvironment (IME) assessment, and susceptibility. The findings establish a basis for increasing the OS of LUAD patients.

## Materials and methods

### Data preparation

The RNA-sequencing expression (level 3) and clinical information of 517 LUAD cases and 59 normal samples were retrieved from The Cancer Genome Atlas (TCGA) via the Genomic Data Commons (GDC) Data Portal (https://portal.gdc.cancer.gov/). The GSE72094 (Schabath et al. 2016) data of 442 LUAD subjects were retrieved from the Gene Expression Omnibus (GEO) (http://www.ncbi.nlm.nih.gov/geo/).

Inclusion criteria: (1) pathologic TNM stage (pTNM_stage) was available; (2) age was available; (3) gender was available; (4) vital status were available; (5) days to death or days to last follow-up were available.

The TCGA LUAD subjects served as the training cohort, while the GSE72094 LUAD subjects were employed as the external validation cohort after conditional filtering was applied.

### Identifying differentially expressed genes (DEGs)

The analysis of DEGs (up-regulated and down-regulated genes) of the LUAD samples in the training cohort and normal samples retrieved from TCGA was performed via the online website (https://www.home-for-researchers.com). “Adjusted *p < *0.05 and Log2 (Fold Change) > 1 or Log2 (Fold Change) <  − 1” denoted the threshold for the DEGs.

The hallmark mitotic spindle gene set was obtained from MSigDB (https://www.gsea-msigdb.org/gsea/msigdb/). Batch survival analysis of the hallmark mitotic spindle genes in LUAD (340 cases) were performed using an online website (https://www.home-for-researchers.com). The survival differences between tumor and normal groups were assessed using the Kaplan–Meier (KM) survival analysis with the log-rank test. Log-rank tests and univariate Cox proportional hazards regression were employed to determine *p* values and hazard ratio (HR) with a 95% confidence interval (CI) for KM curves.

Finally, we toke an intersection of analyzed hallmark mitotic spindle genes and DEGs (up-regulated genes). The protein–protein interaction (PPI) network was constructed using an online website (https://cn.string-db.org/) (Szklarczyk et al. 2023), and visualized by Cytoscape (v3.9.1).

### Risk score model construction

The online website (https://www.home-for-researchers.com) was used to normalize the data log2 (TPM + 1) and maintain samples (340 LUAD cases) with clinical information simultaneously. The lambda and risk score were calculated using the least absolute shrinkage and selection operator (LASSO) regression algorithm. All of the 340 patients were classified into high and low-risk groups based on the median risk score, and the differences in OS between the two groups were analyzed using the log-rank test.

### Construction and validation the nomogram model

Univariate and multivariate cox regression analyses were performed to identify the appropriate terms for constructing the nomogram with the following predictors: age, gender, pTNM_stage, and risk score. A nomogram was constructed based on the results of multivariate cox proportional hazards analysis and it was employed to predict the 1-year, 3-year, and 5-year OS. The nomogram graphically represented the factors for calculating points with each risk factor in the “rms” R package to predict the OS in a patient. The predictive ability of the nomogram was evaluated by calculating Harrell’s concordance index (C-index). The plotted calibration curves were used to assess and validate the nomogram. Time-dependent receiver operating characteristic (tROC) curves were used to assess the prognosis sensitivity and specificity at 1-year, 3-year, and 5-year intervals. The area under the curve (AUC) value of the ROC curve was computed to predict the accuracy using the “time ROC” R package.

The accuracy of the nomogram model was validated using GSE72094 data. Depending on the median risk score, two groups of high and low expression for seven genes were identified based on their level of expression. The OS of patients in the two groups was compared using the log-rank test. The risk score and clinical data were merged to validate the prediction model using the “rms” and “time ROC” R packages.

### Immune microenvironment analysis

The immune score of tumor and normal groups were calculated using the EPIC algorithm with the “immunedeconv” R package. The results were implemented and displayed using the “ggplot2” and “pheatmap” R packages.

The “ggplot2” and “pheatmap” R packages were employed to determine the gene expression values for the eight immune checkpoints, including Sialic Acid Binding Ig Like Lectin 15 (SIGLEC15, CD33L3), Cytotoxic T-Lymphocyte Associated Protein 4 (CTLA4, CD152), Lymphocyte Activating 3 (LAG3, CD223), PD-1 (CD279), T Cell Immunoreceptor With Ig And ITIM Domains (TIGIT), CD274 (PD-L1), Hepatitis A Virus Cellular Receptor 2 (HAVCR2, TIM3), and Programmed Cell Death 1 Ligand 2 (PD-L2, CD273) in the training cohort.

The “ggstatsplot” and “pheatmap” R packages were employed to highlight the correlation between seven-gene expression and immune score and immune checkpoints, respectively. Spearman correlation analysis was used to assess the correlation between quantitative variables with non-normal distribution.

Bioinformatics analysis revealed that the expression of seven-gene was related to the prognosis of LUAD. The effect of TOP2A, PLK1, ANLN, LMNB1, and ECT2 on LUAD occurrence and progression has been demonstrated in vitro and/or in vivo (Kou et al. 2020; Shin et al. 2020; Xu et al. 2019; Li et al. 2022a; Kosibaty et al. 2021). As such, we only validated the protein expression of KIF15 and DLGAP5 using western blot.

### Cell lines and cell culture

All cell lines (BEAS-2B, A549, H1299, H1975, and PC-9) were purchased from the cell bank of Chinese Academy of Sciences. All cells were cultured in DMEM supplemented with 10% fetal bovine serum (FBS) containing double resistance (1%). All cell lines were incubated at 37 °C in 5% CO2.

### Western blot

Total protein was extracted from cells by RIPA buffer (Beyotime Biotechnology, China) and quantified using the BCA assay (Beyotime Biotechnology, China). Sample proteins (20 µg/lane) were separated by the 6% SDS-PAGE (Epizyme Biomedical Technology, China) and transferred to the nitrocellulose (NC) membranes. The membranes were blocked for 1 h at room temperature (RT) with 5% nonfat milk and subsequently incubated with the primary antibodies of anti-KIF15 (1:1000; Proteintech, China), anti-DLGAP5 (1:1000; ABclonal Technology, China), and anti-GAPDH (1:3000; Thermo Fisher Scientific, USA) overnight at 4 °C, followed by anti-rabbit secondary antibody (1:5000; Thermo Fisher Scientific, USA) for 2 h at RT. Protein bands were visualized using an enhanced chemiluminescence western blotting detection kit (Thermo Fisher Scientific, USA) and Bio-Rad ChemiDoc (Bio-Rad Laboratories, USA). GAPDH was used as the internal reference.

### Statistical analysis

Student’s *t* test was used to compare the differences between the two groups. All data analyses and R packages were implemented in R version 4.2.1. *p* value < 0.05 denoted statistical significance.

## Results

### Data preparation and DEGs screening

A total of 340 (TCGA) and 393 (GSE72094) LUAD samples were screened out as the training and validation cohorts, respectively. The clinical and expression data from 340 (TCGA) and 393 (GSE72094) LUAD samples were normalized. The clinical characteristics are shown in Table 1.

**Table 1:** Clinical characteristics of TCGA and GSE72094 cohorts

Characteristics	TCGA, *n* = 340, (%)	GSE72094, *n* = 393, (%)
Age	64.85 ± 10.30	69.35 ± 9.50
Gender		
Female	172(50.59)	219(55.73)
Male	168(49.41)	174(44.27)
pTNM_stage		
I	174(51.18)	254(64.63)
II	83(24.41)	67(17.05)
III	61(17.94)	57(14.50)
IV	22(6.47)	15(3.82)
Status		
Alive	203(59.71)	282(71.76)
Dead	137(40.29)	111(28.24)

The genetic information of the training cohort and 59 normal tissues retrieved from the TCGA database were explored for DEGs. A total of 2699 DEGs were screened out, with 965 up-regulated genes and 1734 down-regulated genes.

**Fig. 1:**
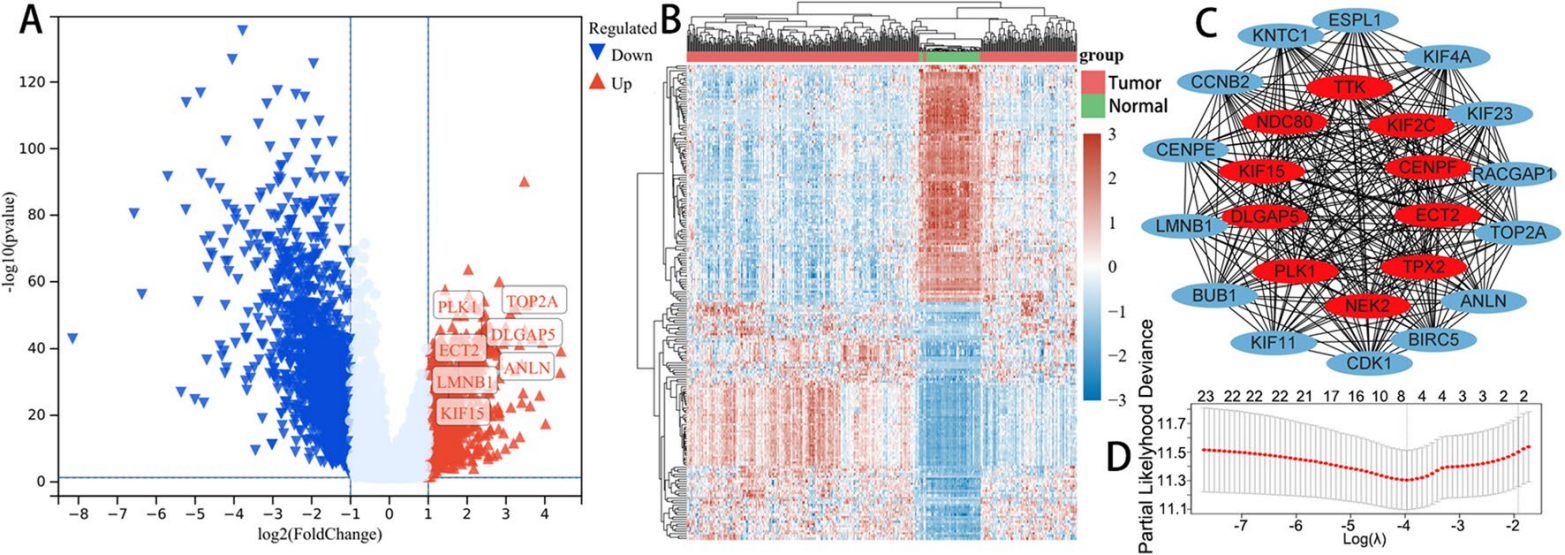
The volcano map (**A**) of differentially expressed genes in the training cohort. The red dots indicate up-regulated genes; blue dots indicate down-regulated genes; grey dots indicate not significant. The heatmap (**B**) of the differential gene expression (top 50 up-regulated and down-regulated genes). The results of PPI network (**C**) and LASSO regression analysis (**D**) of 24 genes

The mitotic spindle gene set, including 199 genes, was retrieved from MSigDB. A batch survival analysis of 199 genes in 340 LUAD cases revealed that 51 genes were significant difference associated with OS. In this view, we intersected 965 up-regulated genes with 51 genes and discovered 24 genes. The results of PPI network and LASSO regression analysis of 24 genes are shown in Fig. 1.

**Fig. 2:**
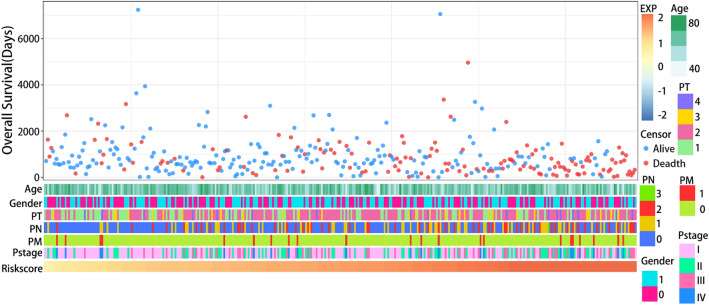
The heatmap of the correlation between risk score, clinical characteristics, and OS

The risk score was calculated using LASSO regression, with 24 genes. Seven genes, including topoisomerase II alpha (TOP2A), polo-like kinase 1 (PLK1), anillin actin-binding protein (ANLN), discs large homolog associated protein 5 (DLGAP5), epithelial cell transforming sequence 2 (ECT2), lamin B1 (LMNB1), and kinesin family member 15 (KIF15), were eventually screened out. Figure 2 depicts the correlation between risk score, clinical characteristics, and OS. The overexpression of seven genes may be related to the carcinogenesis of normal lung cells.

**Fig. 3:**
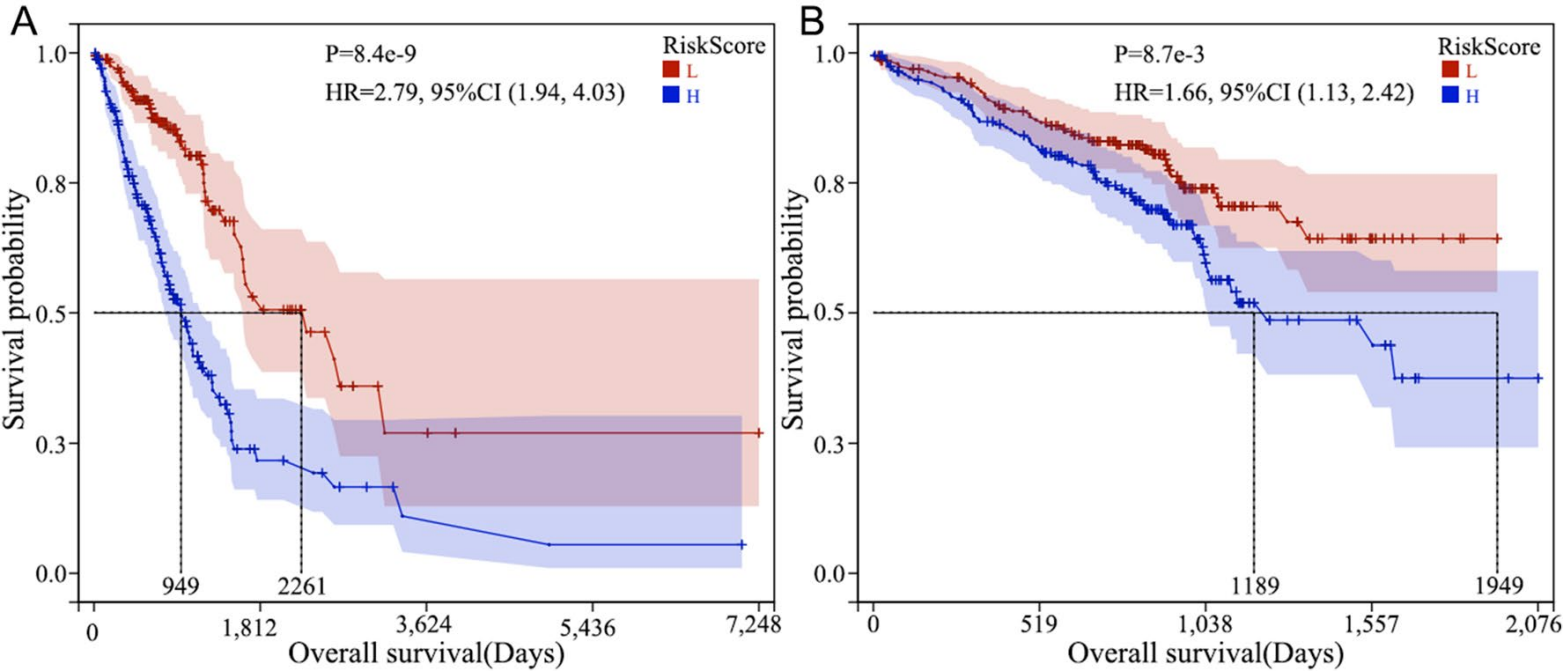
KM curve of OS in high- and low-risk score groups (**A** training cohort; B validation cohort)

The risk score = (− 0.0682) × EXP (TOP2A) + 0.1794 × EXP (PLK1) + 0.2268 × EXP (ANLN) + 0.3342 × EXP (DLGAP5) + 0.0131 × EXP (ECT2) + 0.039 × EXP (LMNB1) + (− 0.4823) × EXP (KIF15). Next, we classified the 340 patients into high and low-risk groups based on their median risk score and performed KM curve analysis. The high-risk group was characterized by a lower OS than the low-risk group; with a statistically significant difference (*p < *0.01). The external validation cohort data verified the OS difference between the high and low-risk groups (*p* = 0.0087). Results are represented in Fig. 3.

### Construction and validation of the Cox and nomogram model

**Fig. 4:**
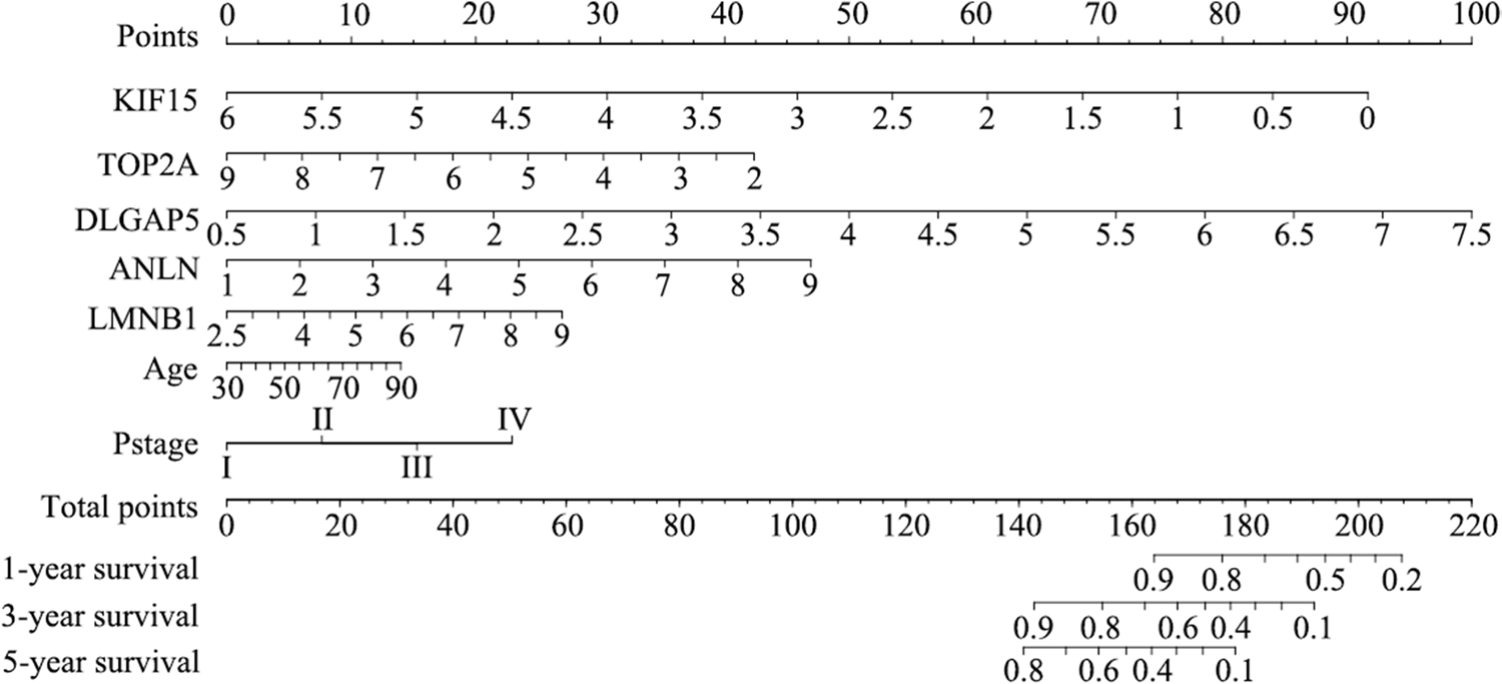
Nomogram prediction model for LUAD based on mitotic spindle seven genes

**Fig. 5:**
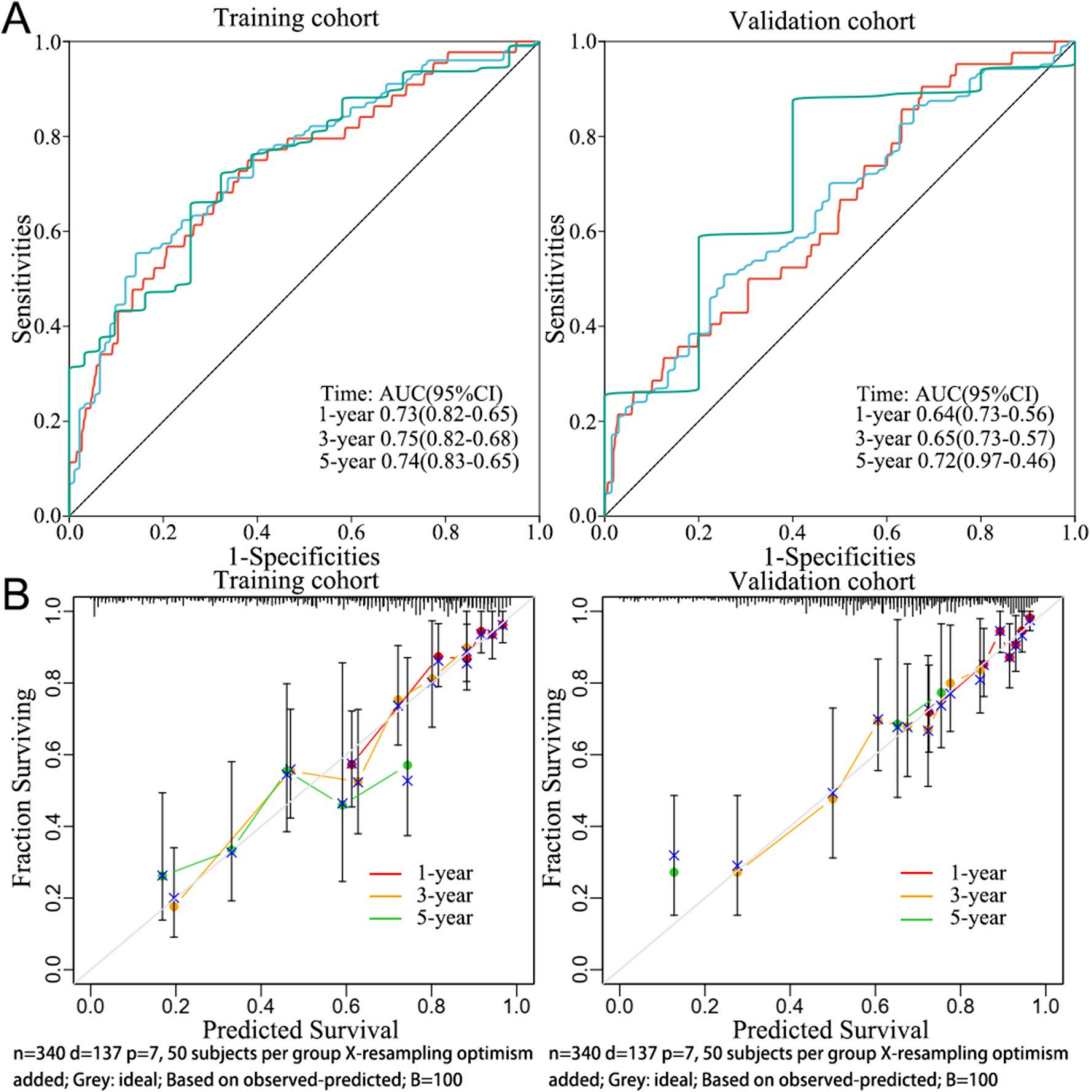
The time-ROC curve (**A**), calibration curve (**B**), and 1-year, 3-year, and 5-year AUC value of nomogram prediction model

A predictive nomogram model was built to investigate the predictive potential of seven genes in LUAD patients. Univariate and multivariate Cox regression analysis with the predictors (age, gender, pTNM_stage, risk score) revealed three predictors (age, pTNM_stage, risk score), which were integrated into the model. The nomogram model is displayed in Fig. 4. The c-index values of the nomogram prediction model were 0.738 (training cohort) and 0.672 (validation cohort). The tROC curves were employed to evaluate the model, and the 1-, 3-, and 5-year AUC values were 0.73, 0.74, and 0.7, respectively. On the other hand, the 1-, 3-, and 5-year AUC values of the validation cohort were 0.63, 0.64, and 0.73, respectively. Figure 5 shows the calibration curves and tROC curves of the training and validation cohorts.

These data demonstrate that the nomogram model based on the 7-gene risk score has good predictive ability in LUAD patients.

### Immune microenvironment analysis

**Fig. 6:**
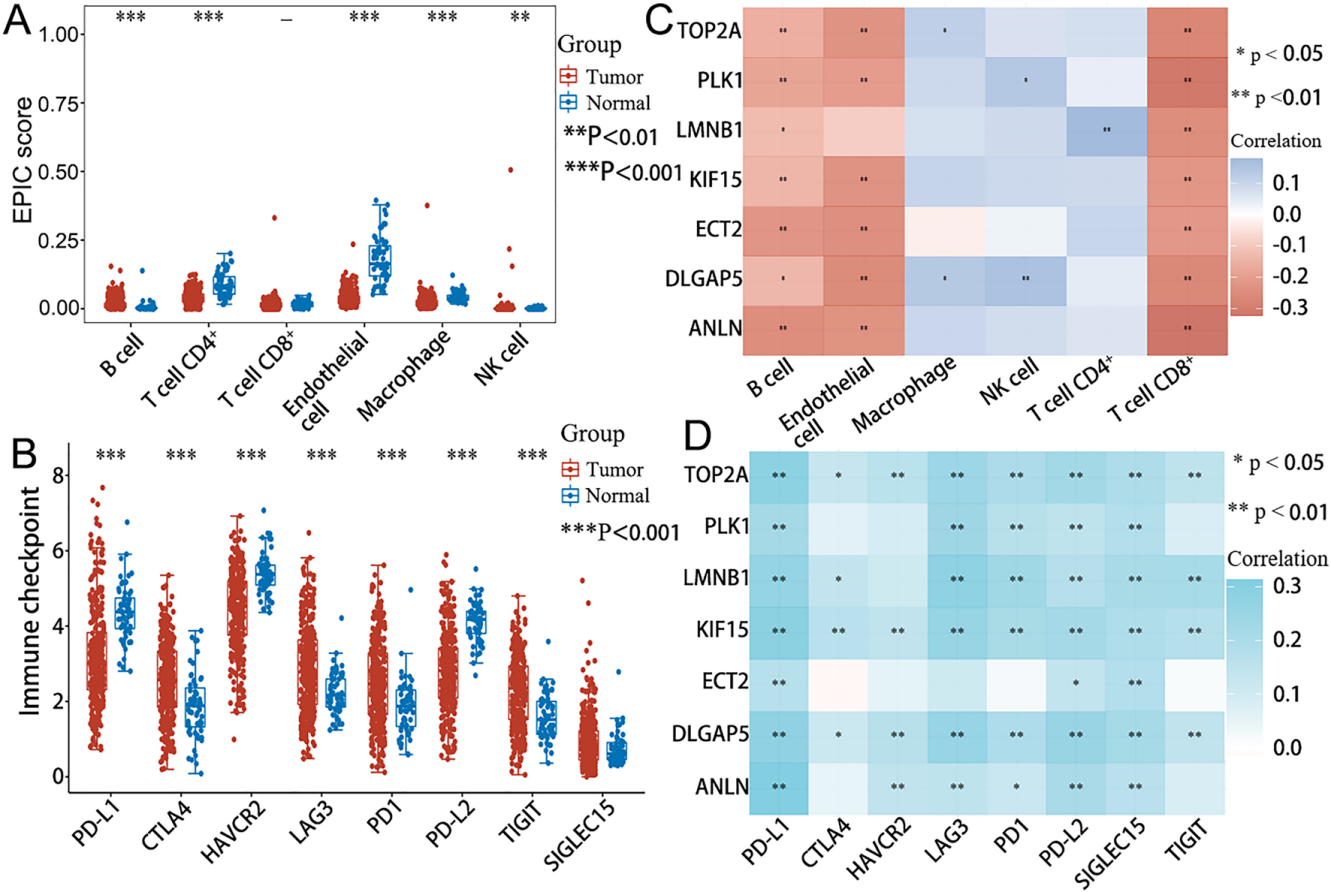
Correlation of training cohort with immune cells (**A**) and immune checkpoints (**B**). Correlation of seven genes expression with immune cells (**C**) and immune checkpoint (**D**)

The immune score results revealed a positive correlation of the training cohort with B cells and NK cells and a negative correlation with T cell CD4 + , Endothelial cells, and Macrophages (*p < *0.05). Immune cell correlation analysis revealed that, the expression of seven genes was negatively correlated with B cell, Endothelial cell (exclude LMNB1), and T cell CD8 + in immune cell correlation analysis (*p < *0.05). Moreover, the training cohort was positively correlated with CTLA4, LAG3, PD-1, TIGIT, and negatively correlated with PD-L1, HAVCR2, and PD-L2 in the immune checkpoint analysis (*p < *0.05). The seven-gene expression was positively correlated with multiple immune checkpoints (*p < *0.05). The results are demonstrated in Fig. 6.

**Fig. 7:**
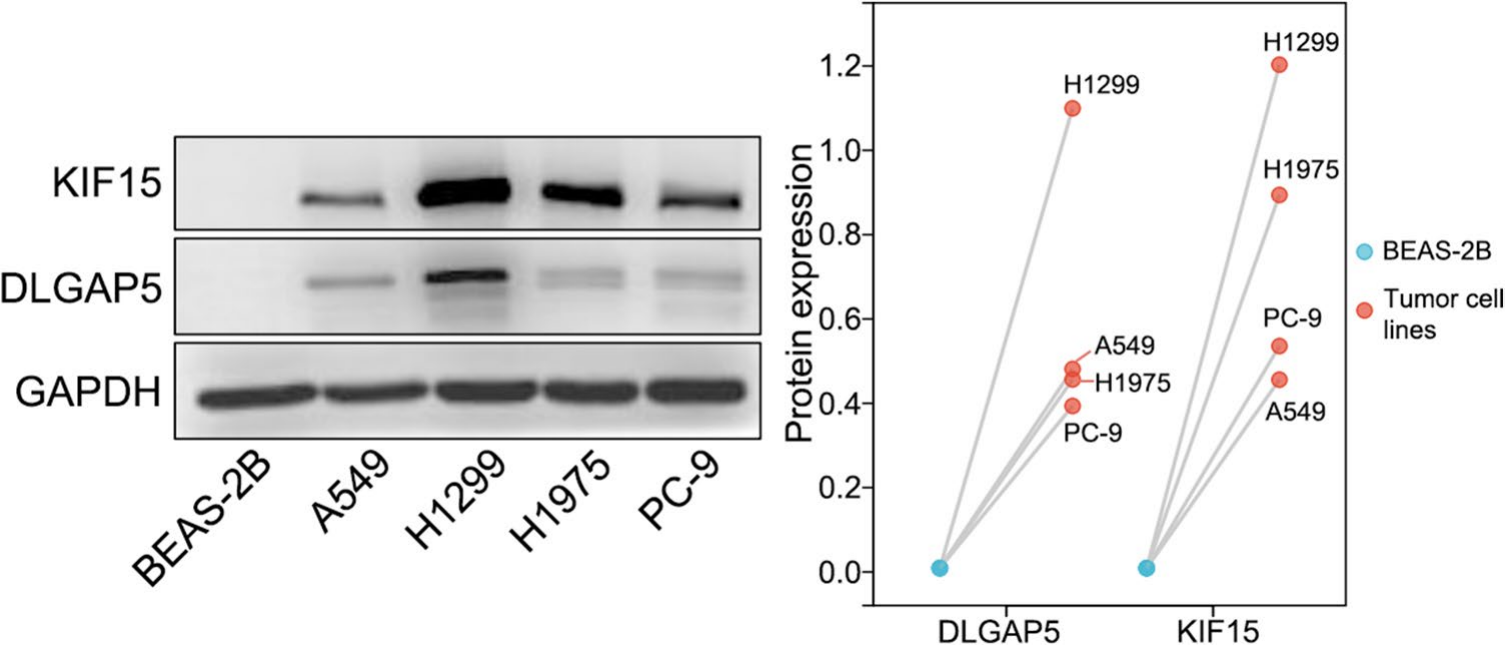
Western blot results of DLGAP5 and KIF15 expression

As revealed in Fig. 7, the expression of DLGAP5 and KIF15 at protein level in A549, H1299, H1975, and PC-9 cell lines was higher than that in BEAS-2B cell line.

## Discussion

Prometaphase marks the beginning of mitosis, which ends with chromosomal segregation in anaphase (Pavin and Tolic 2021). The orientation and location of the spindle determine the relative position of daughter cells and the partitioning of cellular material (Wu et al. 2017). Numerous post-translationally modified proteins play a role in the coordinated assembly and regulation of the mitotic spindle (Ong and Torres 2019; Prosser and Pelletier 2017). The centrosome is the primary microtubule-organizing center in animal cells, and it regulates spindle formation, chromosome segregation, and other processes (Hoffmann 2021). Centrosomes migrate to opposing poles of the mitotic spindle of the nuclear envelope during mitosis to ensure chromosomal segregation by promoting spindle bipolarity (Nigg and Holland 2018). Researchers have progressively found that several eukaryotic cells can complete spindle assembly and cell division without centrosomes (Bettencourt-Dias 2013), but mitosis fidelity of declines (Sir et al. 2013). Many genetic or other disorders have this as one of their causes. Studies on mitotic spindle genes in predicting the prognosis and the IME of LUAD are scarce. Therefore, our current investigation is geared toward finding potential targets for the evaluation and treatment of LUAD.

In our model, we identified age, pTNM_stage, and risk score as prognostic factors in LUAD. The external validation cohort confirmed that the variables were negatively correlated with LUAD prognosis. These findings demonstrate that high seven-gene expression is a poor predictor of LUAD prognosis.

Whilst TOP2A regulates chromosome segregation, DNA topology, and cell-cycle progression (Watt and Hickson 1994, Wang, 2002, Panvichian et al. 2015), its role in the initiation and progression of LC is poorly understood. A mechanistic study demonstrated the role of TOP2A in the PI3K-AKT pathway, regulated by PTEN (Kang et al. 2015). Du and colleagues found up-regulated TOP2A levels in LUAD A549 cells (Du et al. 2020). Elsewhere, TOP2A induced proliferation and metastasis of LUAD (Kou et al. 2020) and was linked to chemotherapy toxicity and survival in NSCLC patients (Grenda et al. 2020). Another study found similar results in patients with small cell lung cancer (SCLC) (Nicos et al. 2021).

PLK1 is critical in different mitotic stages, including centrosome maturation, spindle formation, chromosome segregation, and cytokinesis (Christoph and Schuler 2011). Studies have further highlighted the regulatory role of PLK1 in spindle orientation and astral microtubule formation by regulating LRRK1 phosphorylation (Hanafusa et al. 2015, 2019). On the other hand, PLK1 activation promotes NSCLC metastasis (Shin et al. 2020). Zhang and colleagues discovered that, by downregulating PLK1 expression, miR-593-5p inhibited the proliferation of NSCLC cells (Yan et al. 2020). Reda et al. found that inhibiting PLK1 could increase PD-L1 expression (Reda et al. 2022). These findings lay the groundwork for combining chemotherapy and immunotherapy.

ANLN, which is found on chromosome 7q14.2, encodes 1,125 amino acid proteins (Liu et al. 2022a). ANLN exerts a critical role in cell proliferation, differentiation, adhesion, migration, apoptosis, and cycle progression (Naydenov et al. 2021). Xu et al. discovered that ANLN was linked to cancer cell metastasis in LUAD (Xu et al. 2019). Deng and colleagues also demonstrated that by suppressing ANLN expression, miR-30a-5p inhibited LUAD progression significantly (Deng et al. 2021).

DLGAP5 was discovered as a cell-cycle regulated protein (Bassal et al. 2001) and it plays a crucial role in spindle assembly, kinetochore fiber (K-fiber), stabilization, and chromosomal segregation during mitosis (Tsou et al. 2003; Wong and Fang 2006; Ye et al. 2011). Studies have linked DLGAP5 to poor prognosis in NSCLC patients (Schneider et al. 2017; Shi et al. 2022).

ECT2 is essential for cytokinesis (Hirata et al. 2009) and is crucial for the pathological progression of LUAD (Kosibaty et al. 2021). Justilien and colleagues demonstrated that ECT2 has unique and separable roles in cell transformation and cytokinesis and that nuclear localization of ECT2 is required for the growth of transformed LUAD cells but not for cytokinesis in non-transformed cells (Justilien et al. 2017). Chen et al. found that miR-30a-5p could block the activity, migration, and invasion of LUAD cells through targeted inhibition of ECT2 (Chen et al. 2021).

LMNB1 is a constituent protein of the nuclear skeleton (Dechat et al. 2008) that regulates cell proliferation and senescence, chromosome distribution and aggregation, DNA replication, and DNA damage repair (Camps et al. 2015). LMNB1 has been identified as a tumor promoter in LUAD (Tang et al. 2021); it inhibits LUAD cell proliferation by inducing DNA damage and cell senescence (Li et al. 2022a). Li et al. found that LMNB1 potentially regulates LUAD cell proliferation via the AKT pathway (Li et al. 2020).

KIF15, a member of the kinesin-12 family, regulates abnormal cell proliferation, tumorigenesis, and tumor invasiveness (Yu et al. 2019). Downregulation of KIF15 expression by inhibiting Raf/MEK/ERK signaling blocks NSCLC tumorigenesis (Luo et al. 2022). KIF15 variants are also linked to Idiopathic Pulmonary Fibrosis susceptibility (Zhang et al. 2022). There is, however, no consensus on the role of KIF15 in the development and progression of LUAD.

Although the high expression of DLGAP5 and KIF15 in non-small cells indicates a poor prognosis. However, no related studies have validated in LUAD and our WB results support our prediction.

Analysis of the immune score indicates that tumor-infiltrating B cells in LUAD have tumor-regulatory or inhibitory effects (Paijens et al. 2021). The high seven-gene expression can deplete T cell CD8 + , prompting LUAD immune escape. Furthermore, the negative correlation between the seven genes and endothelial cells is linked to distant tumor metastasis (Maishi and Hida 2017). Nonetheless, more experiments are warranted to prove the correlation mechanism.

Tumor antigens may trigger immune cell responses. Immune cells cannot effectively eradicate cancer cells, because they are either dysfunctional or depleted in the tumor environment. TIGIT ligands include Poliovirus Receptor (PVR, CD155), nectin-2 (CD112), nectin-3 (CD113), and nectin-4 (PRR4, PVRL4). TIGIT have a higher affinity to PVR than the others. Nectin-4 is expressed in LC (Challita-Eid et al. 2016), and it is the only nectin family ligand that interacts with TIGIT (Reches et al. 2020). Blocking nectin4-TIGIT interaction can increase the killing effect of immune cells on the tumor in vitro and in vivo (Reches et al. 2020). A primary and follow-up analysis of a randomized double-blind phase II study (CITYSCAPE) found that anti-TIGIT antibody (tiragolumab) plus atezolizumab improved progression-free survival (PFS) (5.4 vs 3.6 months, *p* = 0.015) and objective response rate (ORR) (31.3% vs 16.2%, *p* = 0.031) compared with placebo plus atezolizumab (Cho et al. 2022). However, there is controversy on why the ORR of atezolizumab is 40.2% in the IMpower110 trial (Jassem et al. 2021). In addition, a trial of tiragolumab in combination with atezolizumab in chemotherapy-naïve patients with locally advanced or metastatic NSCLC is underway (NCT03563716). As a result, more evidence is needed to support the effect of anti-TIGIT in NSCLC. A phase I/II clinical trial of recombinant anti-PD-L1 and anti-TIGIT bispecific antibody drug (HLX301) has been completed, and its effect on NSCLC is promising. More research is needed to validate the therapeutic effect of the anti-TIGIT antibody in LUAD.

Furthermore, one mechanism of tumor immune escape is the expression of PD-L1 bind to the PD-1, which inhibits kinases involved in T cell activation (Bally et al. 2016; He et al. 2015). Intriguingly, because of the low PD-L2 expression, the affinity of PD-L2 to PD-1 is 6 times higher than that of PD-L1, indicating that PD-L1 is the primary ligand (Dai et al. 2014).

Anti-CTLA4 is widely used in the treatment of melanoma because it blocks the inhibitory signal involving CTLA4 molecules between antigen-presenting cells and T lymphocytes (Beavis et al. 2018). In a retrospective study, the PFS of NSCLC in the stereotactic body radiation therapy (SBRT) combined anti-PD-1 group was better than that in the SBRT combined anti-CTLA4 group at 18 months (*p* = 0.02), but the OS was comparable between the two groups at 18 months (*p* = 0.08)(Chen et al. 2020). A previous investigation discovered that PVR is a potential predictor or marker of anti-CTLA4 immune response in non-small cell lung cancer (You et al. 2020). Studies on anti-CTLA4 in the treatment of LC are scarce, and more research is warranted to determine its efficacy in LUAD.

LAG3 and CD4 have high structural homology and affinity for MHC class II (Andrews et al. 2017). Galectin-3, LSECtin, and alpha-synuclein fibers are some of their other ligands. LAG3 is primarily expressed in activated T and NK cells. In most cases, LAG3 is found to be co-expressed with PD1, both of which cause T cell exhaustion (Puhr and Ilhan-Mutlu 2019). Therefore, many clinical trials are focusing on the combination of LAG-3 and PD-1/PD-L1 inhibitors, as well as the LAG-3/PD-L1 bispecific antibody. However, research on neoadjuvant or adjuvant therapy for NSCLC or LUAD is limited. The DATAR study found an intriguing phenomenon that LAG-3 overexpression was negatively correlated with survival benefits in NSCLC patients treated with PD-1 axis blockers (Datar et al. 2019). LAG3 could potentially be used to screen patients for immunotherapy.

In the present investigation, while PDL1 and PDL2 expression in the tumor group was lower than that in the normal group, the expression of TIGIT, CTLA4, LAG3, and PD1 in the tumor group was higher than that in the normal group. Immunotherapy of PD-1, PD-L1, and CTLA4 has been widely used in clinical practice. This could imply that these genes can be used to predict or participate in immune regulation. Clinical trials of TIGIT and LAG3 are underway. It is worth noting that six of the seven genes (TOP2A, PLK1, ANLN, DLGAP5, LMNB1, KIF15) were nearly positively correlated with eight immune checkpoints. This could imply that these genes potentially participate in immune regulation and are promising predictive markers for LUAD. However, additional studies or trials are needed to verify these findings.

In a nutshell, this study demonstrates a positive correlation between the high expression of seven genes and poor prognosis of LUAD, and that the seven genes are promising immunotherapy targets.

## Data Availability

The datasets used and/or analyzed during the current study available from the corresponding author on reasonable request.
